# Supplementary material for: A peptide-based viral inactivator inhibits Zika virus infection in pregnant mice and fetuses
Source: Nat Commun. 2017 Jul 25;8:15672. doi: 10.1038/ncomms15672 (PMC5537589; doi:10.1038/ncomms15672)
Supplement: Supplementary Information — Supplementary Figures and Supplementary Table. [file ncomms15672-s1.pdf]

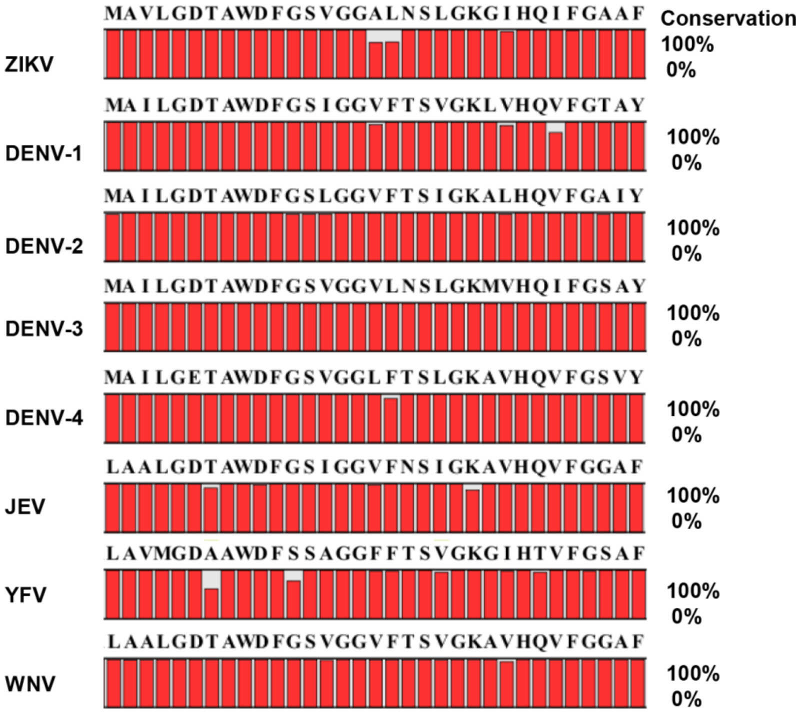

Supplementary Figure 1: Sequence alignment of partial stem region of flaviviruses E prtoeins. Polyprotein sequences of viruses were downloaded from GenBank and aligned by CLC Sequence Viewer software. ZIKV, Zika virus (*n* = 103); DENV, dengue virus (DENV-1, *n* = 48; DENV-2, *n* = 47; DENV-3, *n* = 52; DENV-4, *n* = 50); JEV, Japanese encephalitis virus (*n* = 76); YFV, yellow fever virus (*n* = 80); WNV, West Nile virus (*n* = 814).

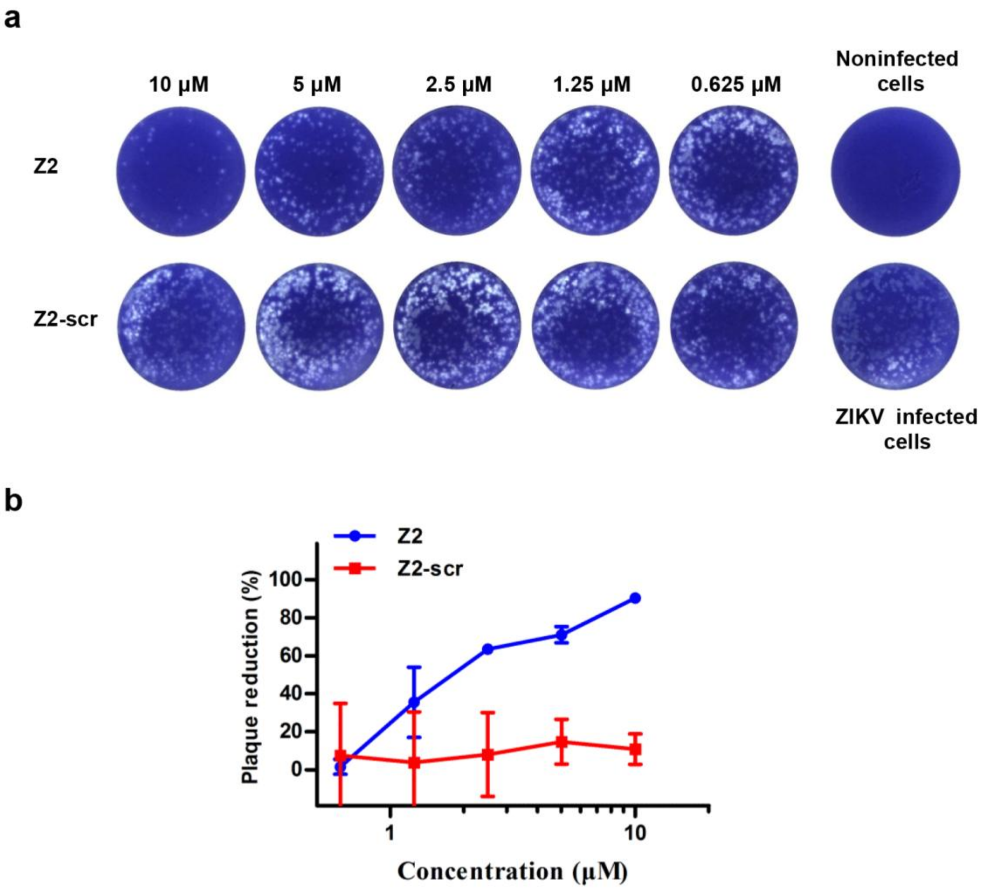

Supplementary Figure 2: Plaque reduction assay to determine ZIKV infection in BHK21 cells. (a) Plaque reduction assay for Z2. ZIKV was incubated with Z2 or Z2-scr in different concentrations for 1.5 h. Then the mixture was added to BHK21 cells seeded in 6-well plates. After 1.5 h, the viral inoculum was removed and 2.5 ml DMEM with 2% FBS and 1% low melting-point agarose overlaid the cells. Four to five days later, the cells were fixed with 4% PFA and stained with 1% crystal violet overnight. **(b)** Plaque reduction curves. Plaques were counted and percentage of plaque reduction was calculated. Data are means ± s.d. of triplicate experiments.

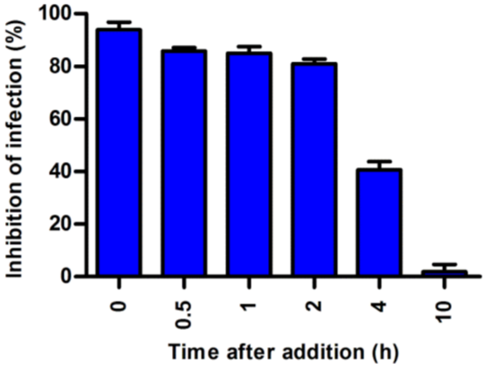

Supplementary Figure 3: Time of addition test to analyze the inhibition of Z2 in ZIKV replication. Z2 was added to cells immediately (0 h) or at various time points (0.5, 1, 2, 4, 10 h) of post infection. 24 h later, the culture medium was changed by DMEM with 2% FBS. About 8 days later when the ZIKV-induced cytopathic effects became evident, the antiviral efficacy was measured by Cell Counting Kit-8. Data are means ± s.d. of triplicate experiments.

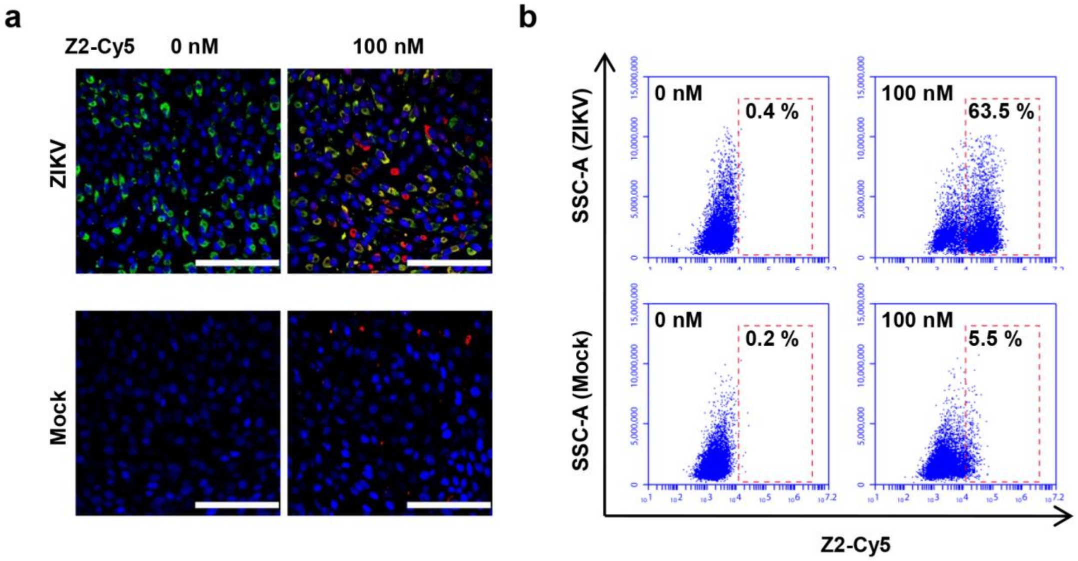

Supplementary Figure 4: Binding of Z2-Cy5 to ZIKV-infected BHK21 cells. (a) Immunofluorescence staining assay. Green, ZIKV E protein; Red, Z2-Cy5; Blue, nuclei. Scale bar = 100 μm. **(b)** Flow cytometry assay. After the incubation of ZIKV or mock infected BHK21 cells with Z2-Cy5, the cells were washed five times and processed by a BD Accuri C6 Flow Cytometer.

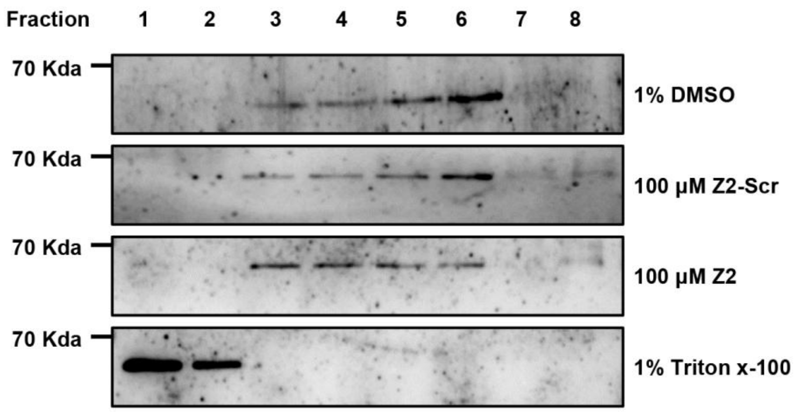

Supplementary Figure 5: Analysis of E protein of ZIKV in the fractions from sucrose density gradient assay. ZIKV was treated with PBS containing 1% (v/v) DMSO, 100 μM Z2 or Z2-scr in PBS containing 1% (v/v) DMSO or PBS containing 1% (v/v) Triton X-100 at 37 ℃ for 2 h, and then centrifuged in a sucrose density gradient. Fractions from top to bottom was collected and percent of the total E protein in each fraction was measured by WB.

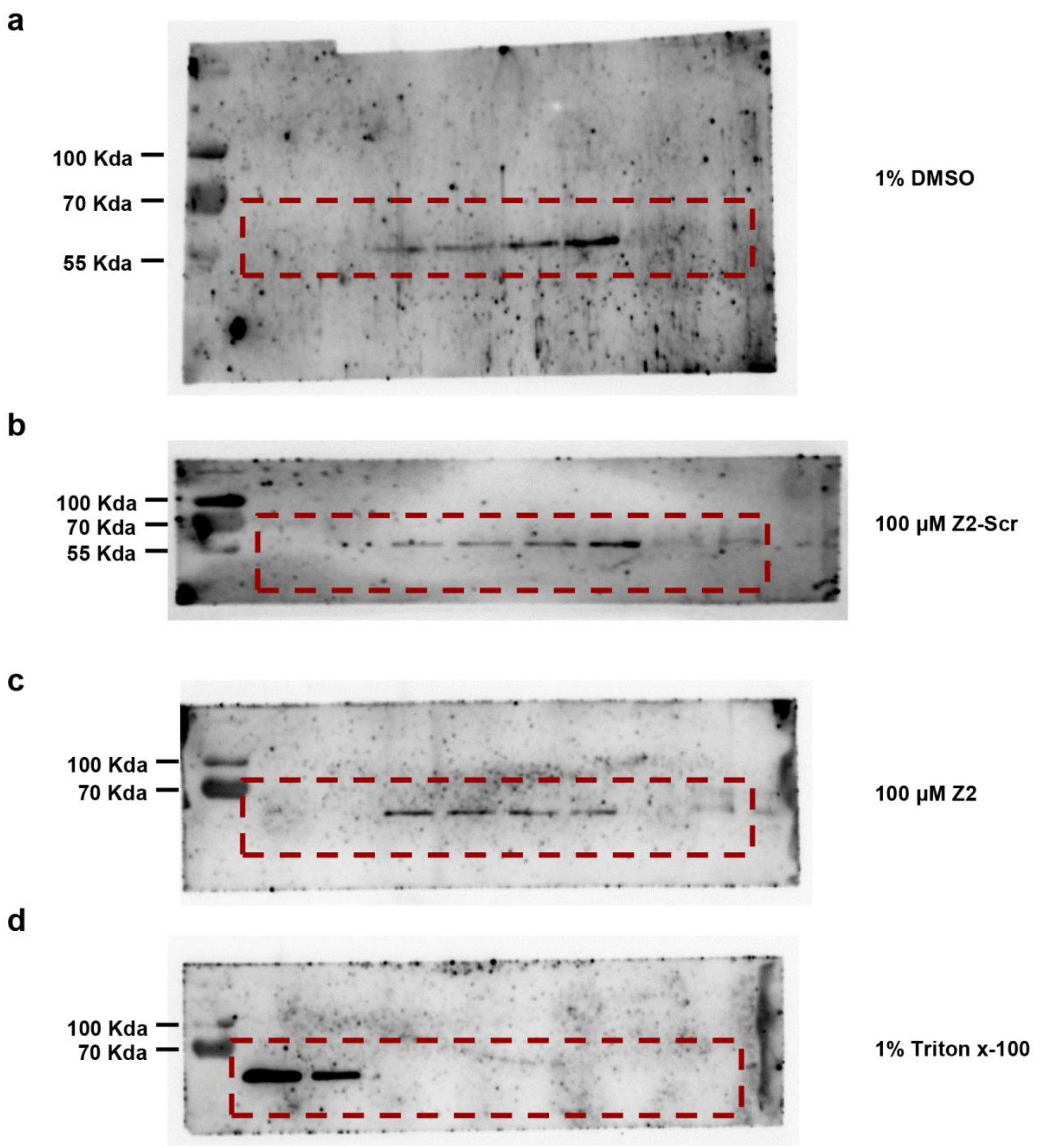

Supplementary Figure 6: Original WB scans for Supplementary Fig. 5. (a) Original WB scans for sample treated with 1% DMSO. **(b)** Original WB scans for sample treated with 100 µM Z2-Scr. **(c)** Original WB scans for sample treated with 100 µM Z2. **(d)** Original WB scans for sample treated with 1% Triton x-100. Areas marked by red box were the corresponding areas used in Supplementary Fig. 5.

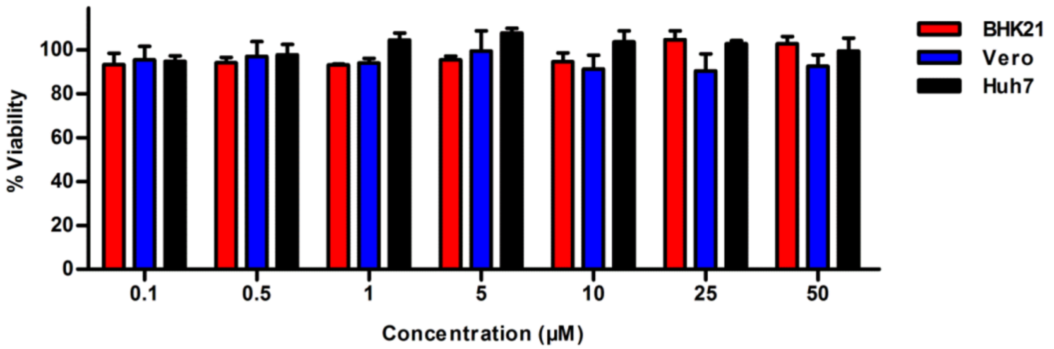

Supplementary Figure 7: Cytotoxicity of Z2 in BHK-21, Vero, and Huh7 cells. Serially diluted Z2 in DMEM with 2% FBS was added to BHK21, Vero, or Huh7 cells for 24 h at 37 ℃. After the solution was removed, the cells were incubated with DMEM containing CCK8 assay solution for 2 h at 37 ℃. Then the absorbance at 450 nm was measured by microplate reader (Infinite M200PRO, Tecan, China). Data are means ± s.d. of triplicate experiments.

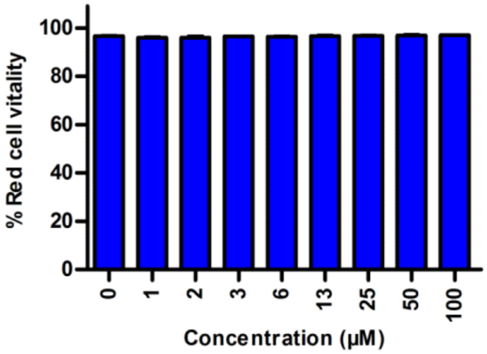

Supplementary Figure 8: Hemoglobin release of mouse red blood cells mediated by Z2. ICR mouse red blood cells (MRBC) were collected to tube with 2.5% citrate sodium through retro-orbital bleeding way. Then MRBC were washed and resuspended in PBS to a final concentration of 2% (v/v). Serially diluted Z2 was added to 2% MRBC in 96 round-well plate, incubated for 1 h at 37 ℃ and centrifuged at 1500 g. The supernatants were migrated to another 96 flat-well plate and the absorbance at 560 nm was measured by microplate reader. Results were normalized against treatment with 1% (v/v) triton X-100 as a control for 100% hemolysis. Data are means ± s.d. of triplicate experiments**.**

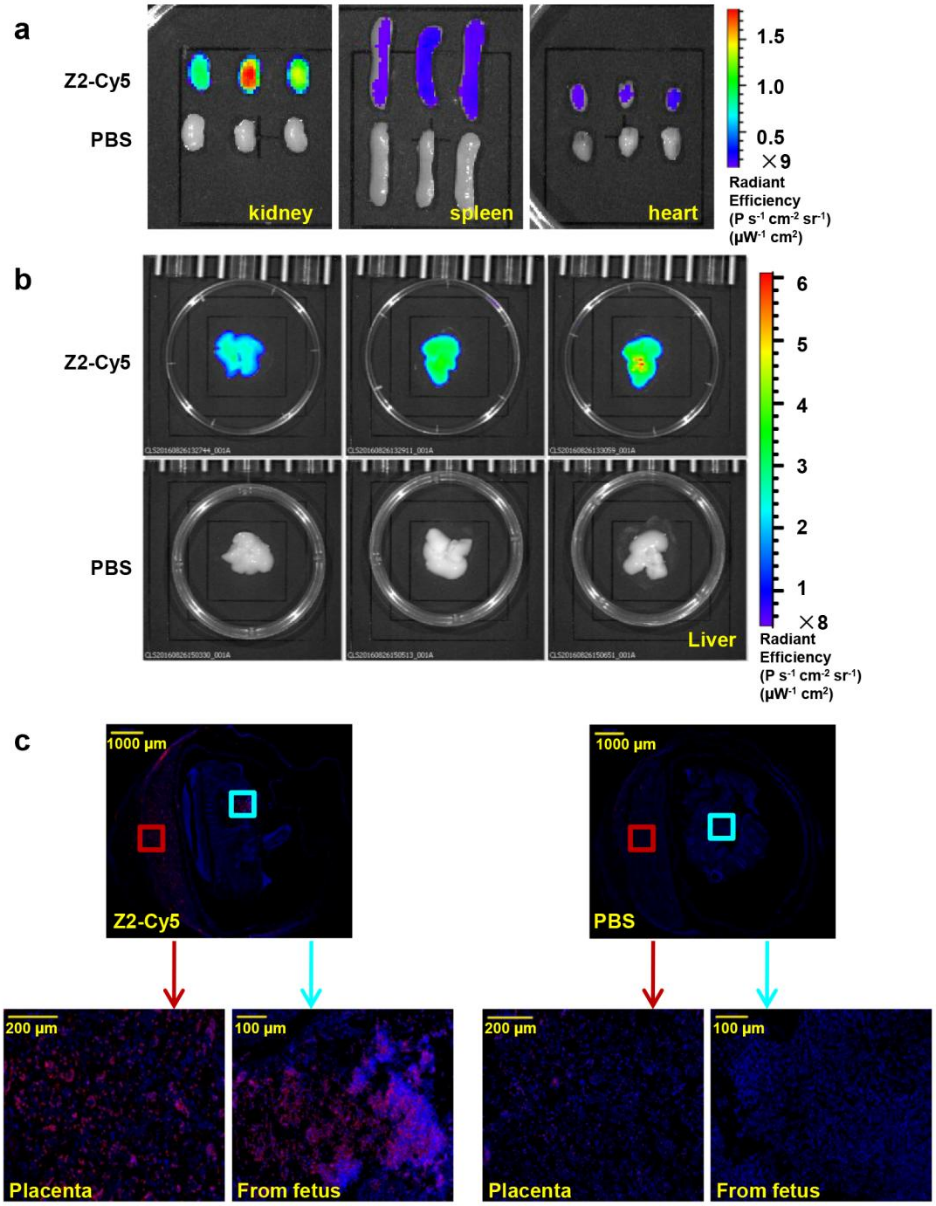

Supplementary Figure 9: Distribution of Z2 in the organs of pregnant ICR mice and their fetuses. (a) Distribution of Z2-Cy5 in the kidney, spleen, and heart. Pregnant ICR mice were treated with Z2-Cy5) (n = 3) or PBS (n = 3) via the intravenous route respectively. 1 h later, distribution of Z2-Cy5 in the organs was monitoring by imaging with the IVIS® Lumina K Series III from PerkinElmer. **(b)** Distribution of Z2-Cy5 in the liver. **(c)** Distribution of Z2-Cy5 in the placenta and fetus.

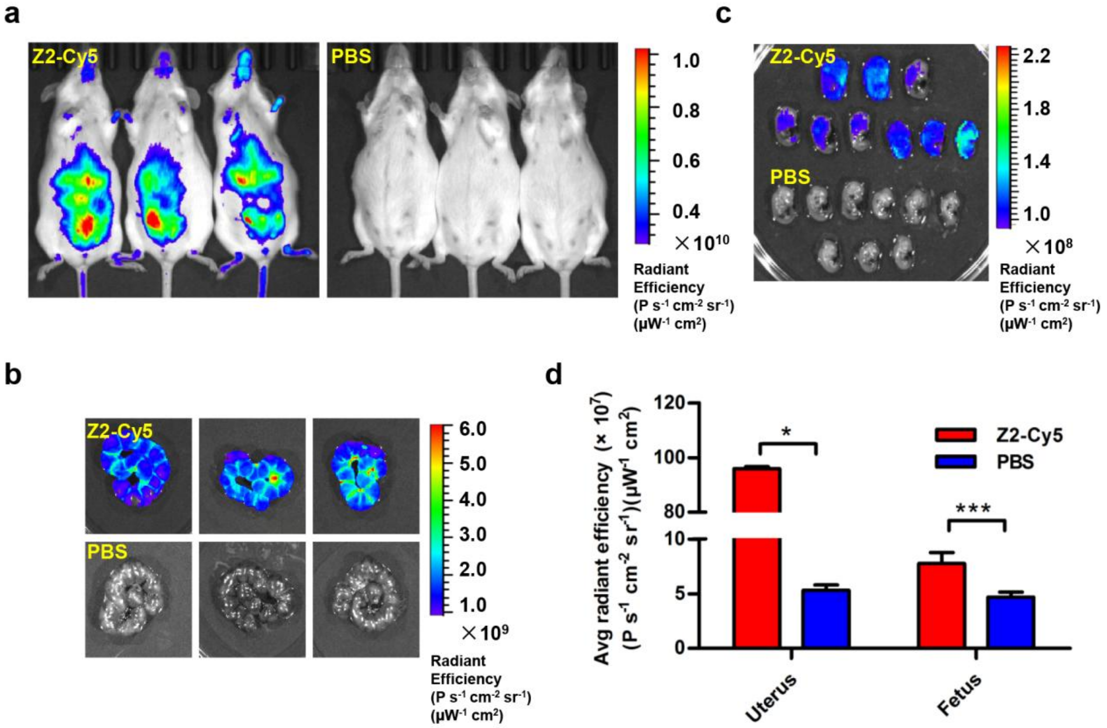

Supplementary Figure 10: Ability of Z2 to penetrate the placental barrier of pregnant ICR mice. (a) Imaging of pregnant ICR mice treated with Z2-Cy5 or PBS by the IVIS ® Lumina K Series III from PerkinElmer. Mice were injected intraperitoneally with 100 μg Z2-Cy5 (n = 3) or PBS (n = 3) as control (for background fluorescence measurement), followed by imaging analysis. (**b**) Imaging of the uteruses from the pregnant mice. **(c)** Imaging of the fetuses (n = 9) removed from uteruses. **(d)** The statistical analysis of results from Supplementary Fig. 10b and10c. Data are means ± s.d. *, p < 0.05; ***, p < 0.001, Student's two-tailed t test.

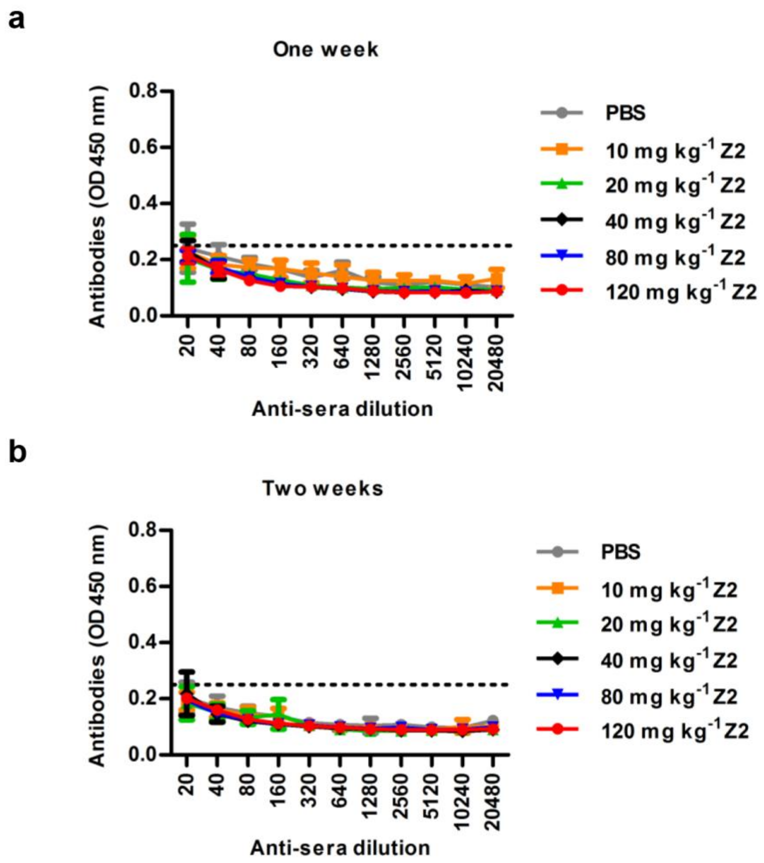

Supplementary Figure 11: Immunogenicity of Z2. (a) Specific antibody response elicited by Z2 in mice at 1 week. **(b)** Specific antibody response elicited by Z2 in mice at 2 weeks. Data are means ± s.d. of triplicate experiments**.**

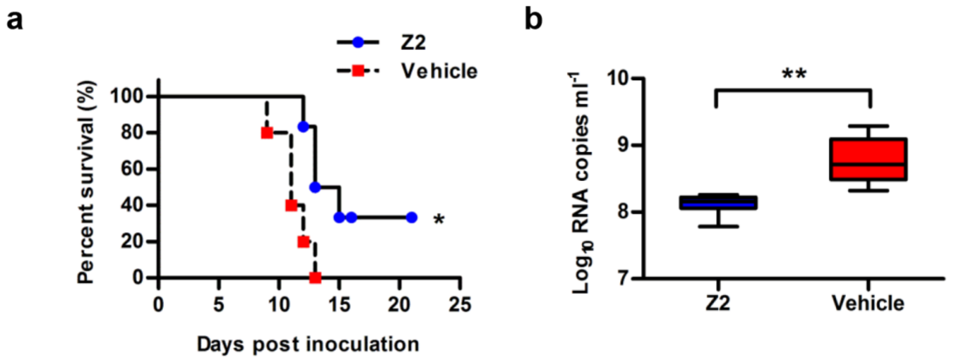

Supplementary Figure 12: Protective activity of Z2 against ZIKV in A129 mice. (a) Survival of ZIKV-infected A129 mice. A129 mice (4 weeks old) were infected with 1×10^5 PFU of ZIKV through intraperitoneal injection (i.p.) route. After 24 h, mice were treated with Z2 (n = 6) at 10 mg kg^{-1} of body weight and vehicle (n = 5) as control. Percentage of mouse survival was observed daily until 21 days post-infection. *, $p < 0.05$, Log-rank (Mantel Cox) test. **(b)** Viral RNA load in sera of ZIKV-infected A129 mice. At day 3 post-infection, mice were retro-orbitally bled to measure viral RNA load in sera by RT-qPCR. Whiskers: 5-95 percentile. **, $p < 0.01$, Mann-Whitney test.

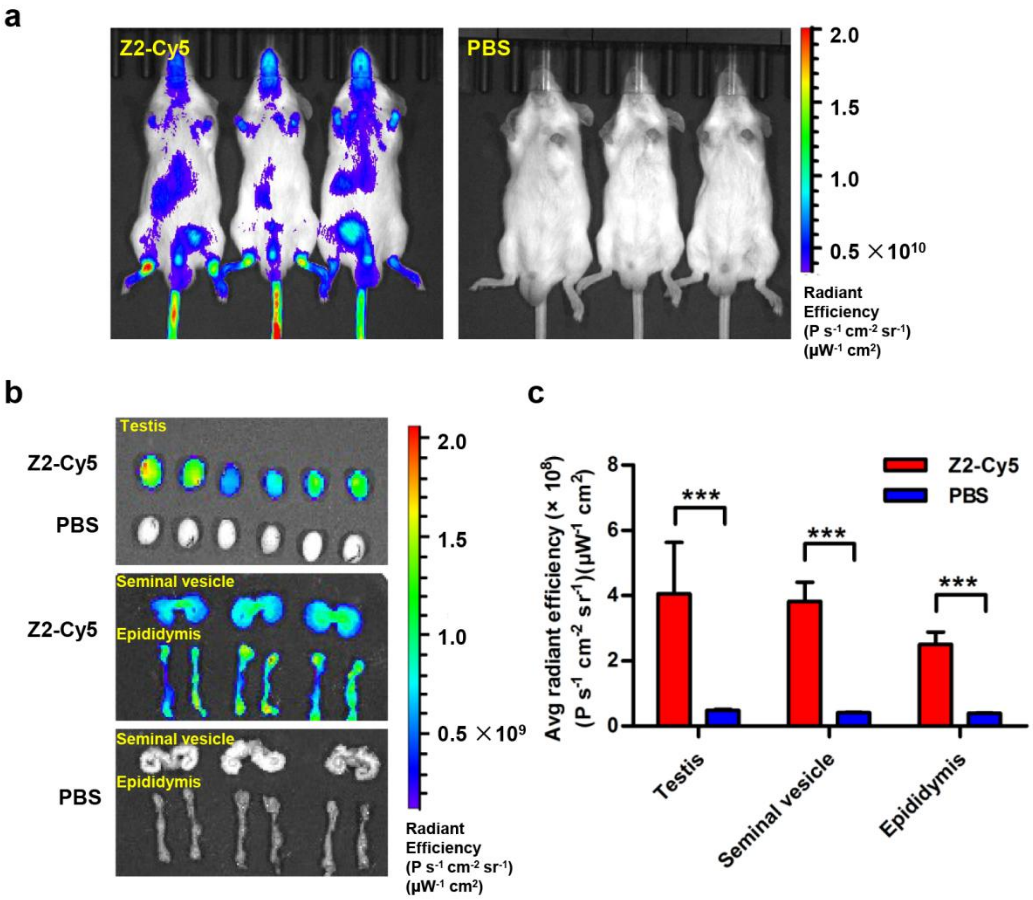

Supplementary Figure 13: Distribution of Z2 in the genital organs of male ICR mice. **(a)** Imaging of male ICR mice treated with Z2-Cy5 or PBS by the IVIS® Lumina K Series III from PerkinElmer. Mice were injected intravenously with 100 μg Z2-Cy5 (n = 3) or PBS (n = 3) as control (for background fluoresce measurement), followed by imaging analysis. (**b)** Imaging of the testis, seminal vesicle and epididymis from the male mice. **(c)** The statistical analysis of results from Supplementary Fig. 13b. Data are means ± s.d.. ***, $p < 0.001$, Student's two-tailed ***t*** test.

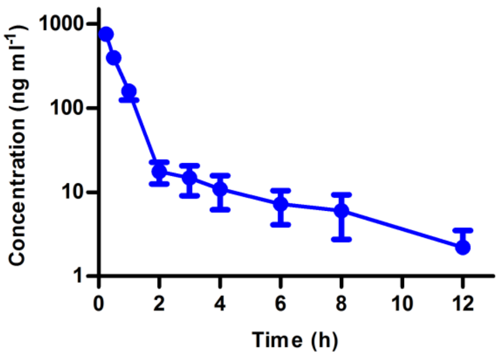

Supplementary Figure 14: Pharmacokinetic study of Z2. SD rats (200 ± 10 g, n = 3) were administrated 10 mg kg^{-1} Z2 intravenously via the tail vein. Blood samples were collected through retro-orbital bleeding at 0.25, 0.5, 1, 2, 3, 4, 8 and 12 h after administration and centrifuged at 3,500 g for 5 min to obtain the serum samples. Then 150 µl methanol containing 15 µg C24M peptide (MTWEEWDKKIEEYTKKIEELIKKS) as an internal standard was added to 50 µl serum to precipitate the protein. After centrifugation at 12,800 g for 10 min, the supernatant was subjected to liquid chromatography-tandem mass spectrometry (LC-MS/MS) analysis, using an AB SCIEX QTRAP 6500 instrument (SCIEX, Boston, USA). All concentration data were dose-normalized and plotted as serum drug concentration time curves. Data are means ± s.d..

Supplementary Table 1: Pharmacokinetic parameters of Z2 in SD rats

**Parameter (unit)**	**Value**
K_{el} (h^{-1})	0.250
$T_{1/2}$ (h)	2.767
T_{max} (h)	0.25
C_{max} (ng ml^{-1})	754.6
AUC $_{(0\text{-}12\ h)}$ (ng ml^{-1}*h)	547.9
AUC $_{inf}$ (ng ml^{-1}*h)	556.7

PK solutions 2.0 (Noncompartmental pharmacokinetics data analysis) was utilized to analyze the pharmacokinetic parameters. K_{el}, elimination constant; $T_{1/2}$, half-life; T_{max}, the time to reach peak drug concentration in serum; C_{max}, peak drug concentration in serum; AUC, Area under the concentration - time curve.
